# Supplementary material for: The Pax transcription factor EGL-38 links EGFR signaling to assembly of a cell-type specific apical extracellular matrix in the Caenorhabditis elegans vulva
Source: bioRxiv. 2024 Sep 6:2024.09.04.611291. Preprint. [Version 1] doi: 10.1101/2024.09.04.611291 (PMC11398461; doi:10.1101/2024.09.04.611291)
Supplement: Supplement 1 [file NIHPP2024.09.04.611291v1-supplement-1.pdf]

bioRxiv preprint doi: https://doi.org/10.1101/2024.09.04.611291; this version posted September 7, 2024. The copyright holder for this preprint (which was not certified by peer review) is the author/funder, who has granted bioRxiv a license to display the preprint in perpetuity. It is made available under aCC-BY 4.0 International license.

Strain	Genotype	Citation
BC10642	*dpy-5(e907) I; sEx10642 (let-653pro*::GFP, *dpy-5+*)	(Hunt-Newbury et al., 2007)
CM2762	*egl-38(gu253[egl-38::gfp]) IV*	(Web Chasser et al, 2019)
ML2358	*noah-1(mc58 [noah-1::sfGFP) I*	(Vuong-Brender et al., 2017)
ML2482	*noah-1(mc68 [noah-1::mCH(int)]) I*	(Vuong-Brender et al., 2017)
OP171	*unc-119(ed3) III; wgIs171 [egl-38::TY1::EGFP::3xFLAG(92C12) + unc-119(+)]*	(Gernstein et al., 2010)
PHX8315	*cutl-18(syb8315[cutl-18::SL2::mCh::H2B]) V*	This paper
PHX8336	*let-653(syb8336 [let-653::SL2::mCherry::H2B]) IV*	This paper
PHX8346	*noah-1(syb8346[noah-1::SL2:mCh::H2B]) I*	This paper
PHX8442	*cutl-18(syb8442[ss::mNeon::cutl-18]) V*	This paper
UP3422	*csIs66 [let-653pro::SfGFP::LET-653(ZP); let-653pro::PH::mCherry] X*	(Cohen et al, 2019)
UP3849	*csIs96 [let-653pro::LET-653(ZP)::SfGFP; lin-48pro::mRFP] II*	(Cohen et al., 2020 PLoS Genetics)
UP3995	*muIs28 [mig-2::GFP; unc-31+]*	(Honigberg and Kenyon, 2000)
UP4011	*noah-1(mc68 [noah-1::mCh(int)]) I; csIs96 [let-653pro::LET-653(ZP)::SfGFP; lin-48pro::mRFP] II*	This paper
UP4129	*noah-1(mc68[noah-1::mCh(int)]) I; csIs96[let-653Pro::LET-653(ZP)::sfGFP; lin-48::pro::mRFP] II; egl-38 (sy294) IV*	This paper
UP4130	*noah-1(mc68 [noah-1::mCh(int)]) I; csIs96 [let-653pro::LET-653(ZP)::SfGFP; lin-48pro::mRFP] II; egl-38(n578) mec-3(n3197) IV*	This paper
UP4162	*lin-1(n304) IV/nT1[qIs51] (IV; V); csIs66 [let-653pro::SfGFP::LET-653(ZP); let-653pro::PH::mCherry] X*	This paper
UP4200	*egl-38(n578) mec-3(n3197) IV; csIs66 [let-653pro::SfGFP::LET-653(ZP); let-653pro::PH::mCherry] X*	This paper
UP4268	*cog-1(sy275) II; csIs66 [let-653pro::SfGFP::LET-653(ZP); let-653pro::PH::mCherry] X*	This paper
UP4291	*unc-32(e189) lin-12(n137n720) III/hT2 [bli-4(e937) let-?(q782) qIs48](I;III); egl-38(gu253[egl-38::gfp]) IV*	This paper
UP4293	*dpy-19(e1259) lin-12(n137) III/hT2 [bli-4(e937) let-?(q782) qIs48](I;III); egl-38(gu253[egl-38::gfp]) IV*	This paper
UP4301	*sur-2(ku9) I; egl-38(gu253[egl-38::gfp]) IV*	This paper
UP4302	*egl-38(n578) mec-3(n3197) IV; cutl-18(syb8315[cutl-18::SL2::mCh::H2B]) V*	This paper
UP4303	*egl-38(n578) mec-3(n3197) IV; cutl-18(syb8442[ss::mNeon::cutl-18]) V*	This paper
UP4310	*noah-1(syb8346[noah-1::SL2:mCh::H2B]) I; egl-38(n578) mec-3(n3197) IV*	This paper
UP4311	*let-653(cs178)IV; cutl-18(syb8442[mNG::CUTL-18]) V; csEx766(lin48pro::LET-653b::sfGFP + myo-2p::GFP)*	This paper
UP4317	*cutl-18(gk516154)(6X outrcoss) V; csIs66 [let-653pro::SfGFP::LET-653(ZP); let-653pro::PH::mCherry] X*	This paper
UP4320	*egl-38(n578) mec-3(n3197) let-653(syb8336 [let-653::SL2::mCherry::H2B]) IV;*	This paper
UP4329	*unc-32(e189) lin-12(n137n720) III/hT2 [bli-4(e937) let-?(q782) qIs48](I;III); cutl-18(syb8442[ss::mNeon::cutl-18]) V*	This paper
UP4338	*dpy-19(e1259) lin-12(n137) III/hT2 [bli-4(e937) let-?(q782) qIs48](I;III); cutl-18(syb8442[ss::mNeon::cutl-18]) V*	This paper
UP4342	*lin-1(n304) IV; wgIs171[egl-38::TY1::EGFP::3xFLAG(92C12) + unc-119(+)]* may contain *unc-119(ed3) III;*	This paper
UP4351	*sur-2(ku9)I; cutl-18(syb8442[ss::mNeon::cutl-18]) V*	This paper

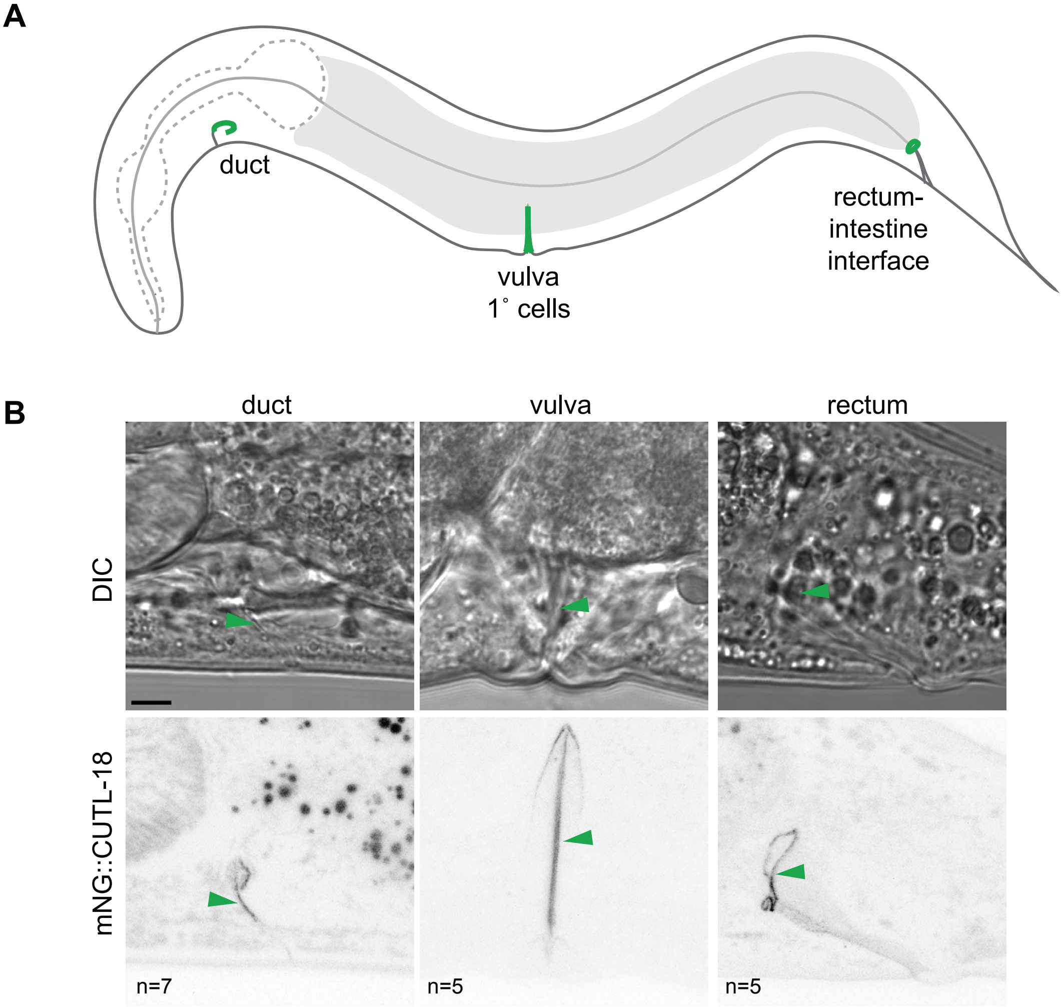
A
duct
vulva
1˚ cells
rectum-
intestine
interface
B
duct
vulva
rectum
DIC
mNG::CUTL-18
n=7
n=5
n=5

bioRxiv preprint doi: https://doi.org/10.1101/2024.09.04.611291; this version posted September 7, 2024. The copyright holder for this preprint (which was not certified by peer review) is the author/funder, who has granted bioRxiv a license to display the preprint in perpetuity. It is made available under aCC-BY 4.0 International license.

Supplement 1: CUTL-18 is a cell-type specific cuticle component in multiple interfacial tubes

A) Diagram of an adult worm. Green indicates the presence of mNG::CUTL-18.

B) mNG::CUTL-18 localization in day 1 adult worms. Top row are DIC images of the indicated tissue. Bottom row are maximum projections of confocal Z stacks through the indicated tissue. Arrowheads point to location of mNG:CUTL-18 in both images. Scale bar 5 μm.

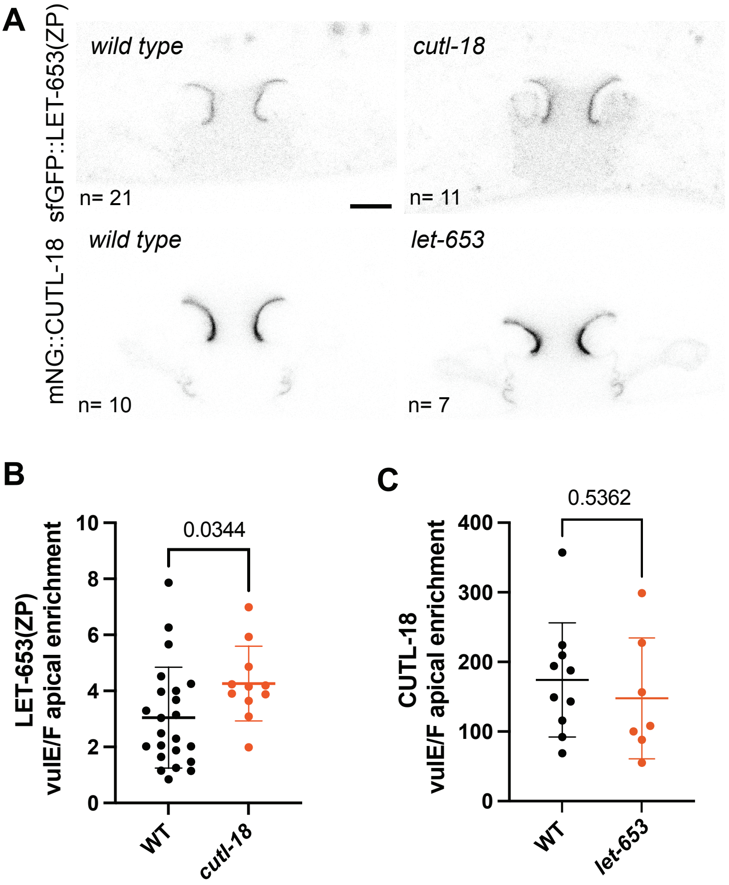
A
sfGFP::LET-653(ZP)
mNG::CUTL-18
wild type
cutl-18
n= 21
n= 11
wild type
let-653
n= 10
n= 7
B
LET-653(ZP) vulE/F apical enrichment
0.0344
10
8
6
4
2
0
WT
cutl-18
C
CUTL-18 vulE/F apical enrichment
0.5362
400
300
200
100
0
WT
let-653

bioRxiv preprint doi: https://doi.org/10.1101/2024.09.04.611291; this version posted September 7, 2024. The copyright holder for this preprint (which was not certified by peer review) is the author/funder, who has granted bioRxiv a license to display the preprint in perpetuity. It is made available under aCC-BY 4.0 International license.

Supplement 2: LET-653 and CUTL-18 do not depend on each other for 1˚ matrix assembly

A) A) Mid- (L4.4/L4.5) L4 larval stage vulvas of wild type, *cutl-18(gk516154)* and *let-653(cs178)* worms expressing LET-653(ZP):sfGFP*(csIs66)* or mNG::CUTL-18. *cutl-18(gk516154)* is a A>T substitution in the splice acceptor site of exon 4 (Thompson et al., 2013), skipping exon 4 would result in a frameshift and premature termination. Outcrossed 6x. *let-653(cs178)* is a null mutation, rescued in the duct by *csEx766*(lin48pro::LET-653b::sfGFP + myo-2p::GFP) (Forman-Rubinsky et al., 2017). Medial confocal slices. Scale bar 5 µm.

B) and C) Apical enrichment of LET-653(ZP) or CUTL-18 on vulE/F cells. See Figure 5F and Methods. P values Kruskal–Wallis test.

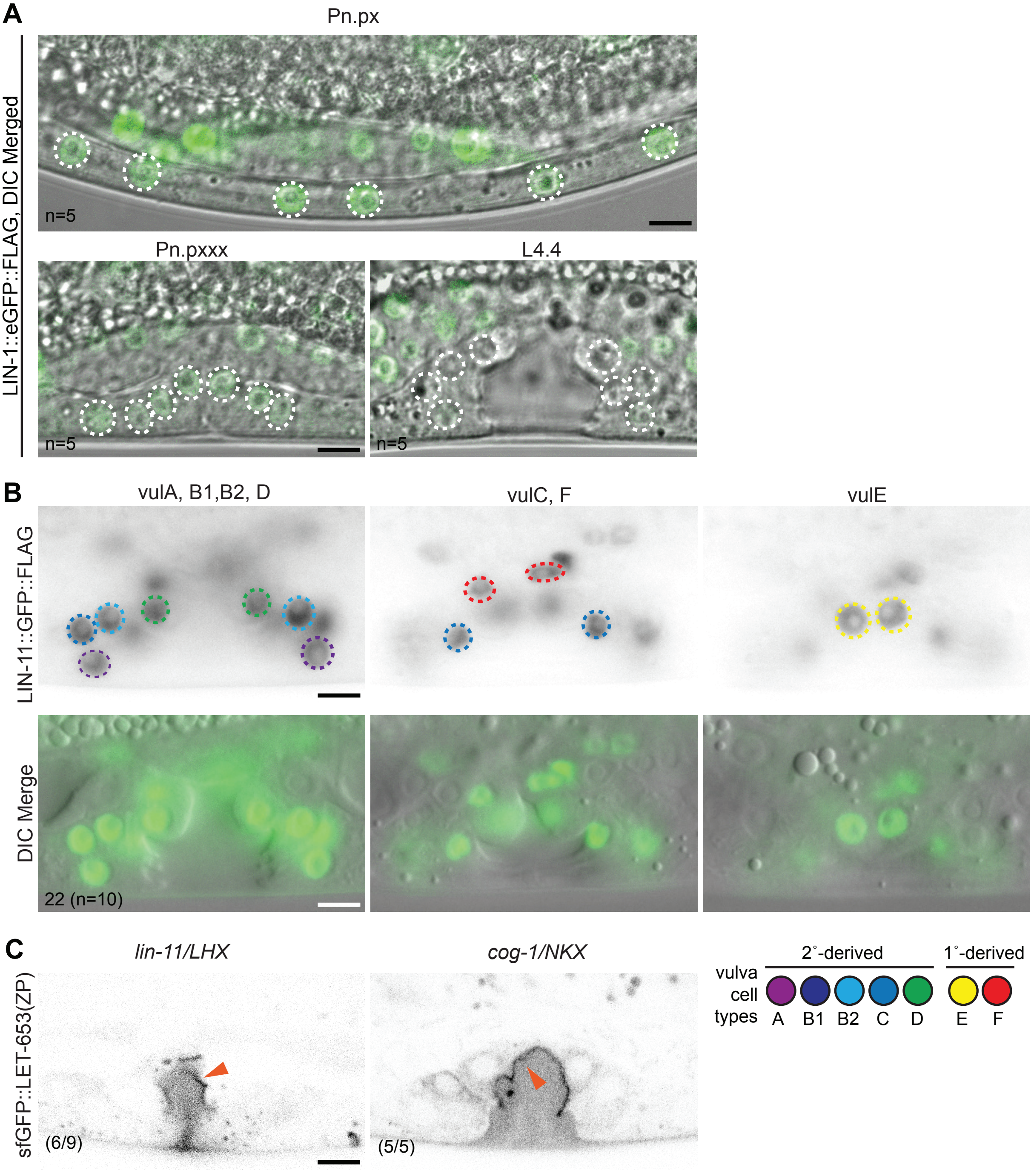
A
Pn.px
LIN-1::eGFP::FLAG, DIC Merged
n=5
Pn.pxxx
L4.4
n=5
n=5
B
vulA, B1,B2, D
vulC, F
vulE
LIN-11::GFP::FLAG
DIC Merge
22 (n=10)
C
lin-11/LHX
cog-1/NKX
sfGFP::LET-653(ZP)
(6/9)
(5/5)
vulva cell types
2°-derived
1°-derived
A
B1
B2
C
D
E
F

bioRxiv preprint doi: https://doi.org/10.1101/2024.09.04.611291; this version posted September 7, 2024. The copyright holder for this preprint (which was not certified by peer review) is the author/funder, who has granted bioRxiv a license to display the preprint in perpetuity. It is made available under aCC-BY 4.0 International license.

Supplement 3: LET-653(ZP) assembly does not require other 1˚ expressed transcription factors.

A) LIN-1::GFP is expressed in all VPC descendants during the L3 stage, but is not detectable in the vulva by L4. DIC + confocal merged images of the VPC descendants (circled) during L3 Pn.px and Pn.pxx, and L4.4 stage.
B) LIN-11::GFP is expressed in all vulva cells. Inverted epifluorescence (top) and DIC + epifluorescence merged images (bottom) of the same L4.4 stage vulva at different focal planes with the above indicated vulva nuclei in focus. Lower left: number of nuclei expressing LIN-11::GFP (total number of mid-L4 (L4.4/L4.5) worms).
C) LET-653(ZP)::sfGFP *csIs66* in mid-L4 vulvas of *lin-11(n389)* and *cog-1(sy295)* worms. LET-653(ZP) enriched on the surface of 1˚ cells (orange arrowhead) Lower left: N/total worms observed. Medial confocal slices at the indicated L4 stages. All scale bars 5 μm.

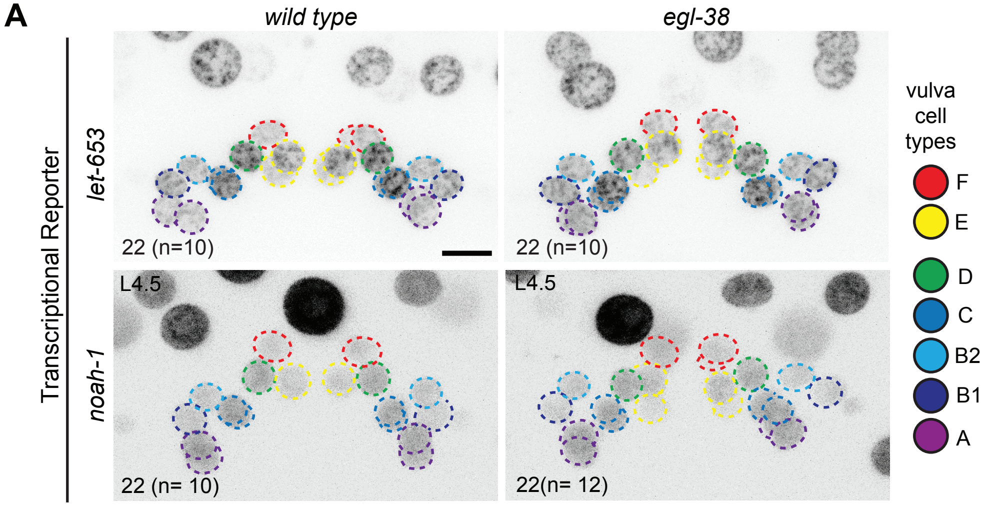
A
wild type
egl-38
Transcriptional Reporter
let-653
noah-1
22 (n=10)
22 (n=10)
L4.5
L4.5
22 (n= 10)
22(n= 12)
vulva
cell
types
F
E
D
C
B2
B1
A

bioRxiv preprint doi: https://doi.org/10.1101/2024.09.04.611291; this version posted September 7, 2024. The copyright holder for this preprint (which was not certified by peer review) is the author/funder, who has granted bioRxiv a license to display the preprint in perpetuity. It is made available under aCC-BY 4.0 International license.

Supplement 4: Transcription of *let-653* and *noah-1* does not require on *egl-38.*

A) Maximum projections of confocal Z stacks through entire vulva of worms expressing the indicated transcriptional reporter. Vulva nuclei are outlined according to the adjacent color code. Scale bar 5 µm Lower left: Number of mCherry positive nuclei, (n= number of worms), wild type Ns represent the same worms as in Figure 2.

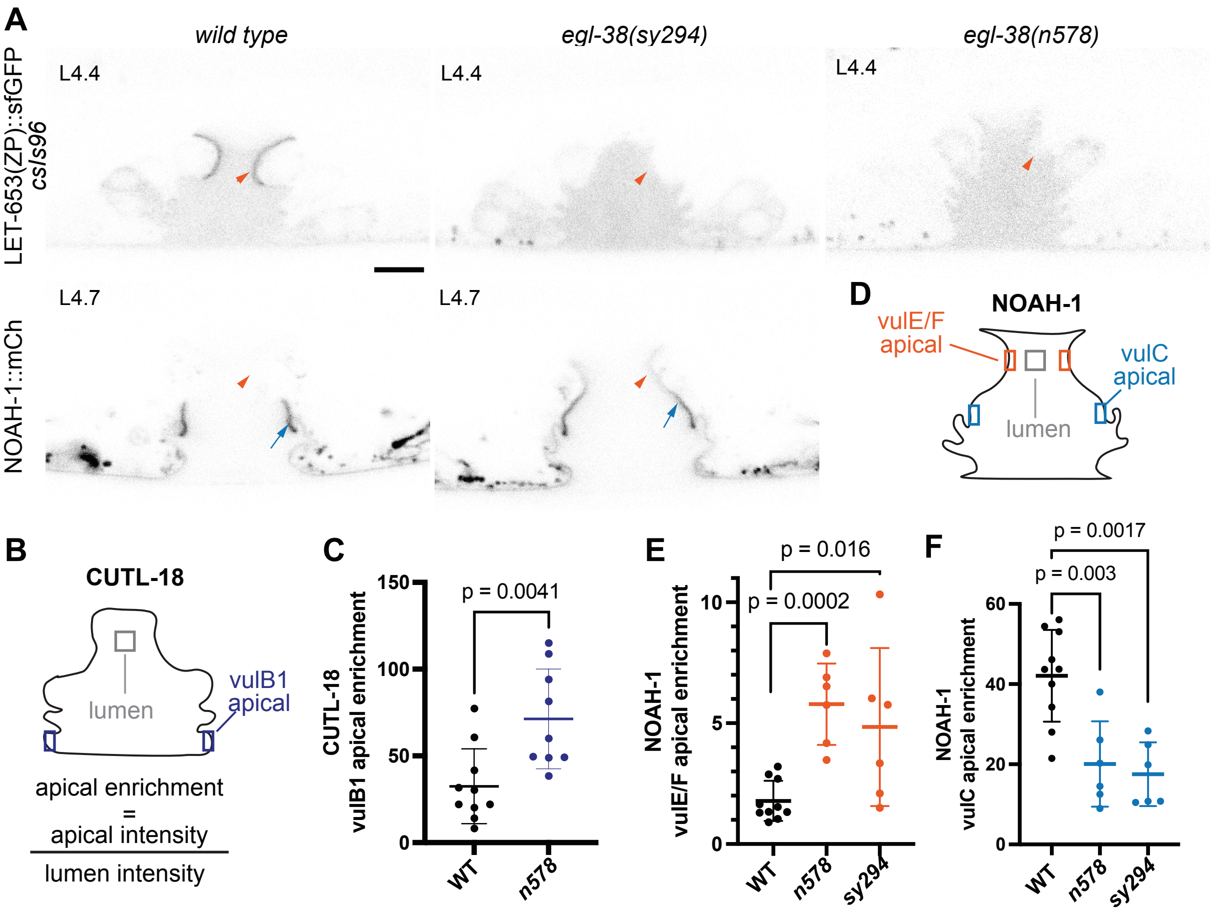

A
wild type
egl-38(sy294)
egl-38(n578)
LET-653(ZP)::sfGFP
csIs96
L4.4
L4.4
L4.4
NOAH-1::mCh
L4.7
L4.7
D
NOAH-1
vulE/F
apical
vulC
apical
lumen
B
CUTL-18
lumen
vulB1
apical
apical enrichment
=
apical intensity
lumen intensity
C
CUTL-18
vulB1 apical enrichment
p = 0.0041
150
100
50
0
WT
n578
E
NOAH-1
vulE/F apical enrichment
p = 0.016
p = 0.0002
10
5
0
WT
n578
sy294
F
NOAH-1
vulC apical enrichment
p = 0.0017
p = 0.003
60
40
20
0
WT
n578
sy294

bioRxiv preprint doi: https://doi.org/10.1101/2024.09.04.611291; this version posted September 7, 2024. The copyright holder for this preprint (which was not certified by peer review) is the author/funder, who has granted bioRxiv a license to display the preprint in perpetuity. It is made available under aCC-BY 4.0 International license.

Supplement 5: EGL-38 requirement for 1˚ matrix is independent of allele and *let-653(ZP)* transgene

A) Vulvas of wild type and *egl-38* mutant worms at the indicated stage expressing LET-653(ZP)::sfGFP (*csIs96)* or NOAH-1:mCherry. As with LET-653(ZP)::sfGFP *csIs66* and *egl-38(n578)* (Figure 5E), in *egl-38* mutants the 1˚ matrix (orange arrowhead) does not contain the proper proteins, while 2˚ cell specific matrices (blue arrows) are maintained. Medial confocal slices at the indicated L4 stages. Scale bar 5 µm

B) Apical enrichment of CUTL-18 quantified by fluorescence intensity at the vulB1 surfaces divided by intensity in a box of the same total size in the lumen. See Methods.

C) Apical enrichment of CUTL-18 on vulB1. Column labels below indicate wild type worms (WT) or *egl-38* alleles (*n578*)*.*

D) Apical enrichment of NOAH-1 quantified by fluorescence intensity at the 2˚descendant vulva cell surfaces, divided by intensity in a box of the same total size in the lumen. See Methods.

E,F) Apical enrichment of NOAH-1 on the indicated the 2˚ descendant vulva cell surfaces Column labels below indicate wild type worms (WT) or *egl-38* alleles (*n578* and *sy294).* All P values Kruskal–Wallis test.
